# Metformin inhibits hepatocellular carcinoma development by inducing apoptosis and pyroptosis through regulating FOXO3

**DOI:** 10.18632/aging.203464

**Published:** 2021-09-21

**Authors:** Zetian Shen, Han Zhou, Aomei Li, Tiancong Wu, Xiaoqin Ji, Lei Guo, Xixu Zhu, Dagan Zhang, Xia He

**Affiliations:** 1 The Affiliated Cancer Hospital of Nanjing Medical University and Jiangsu Cancer Hospital and Jiangsu Institute of Cancer Research, Nanjing 210009, Jiangsu, China; 2 Department of Radiation Oncology, Jinling Hospital, Medical School of Nanjing University, Nanjing 210002, Jiangsu, China; 3 Poolingmed Institute of Medicine, Hangzhou 310016, Zhejiang, China; 4 Guangdong Key Laboratory of Biomedical Measurements and Ultrasound Imaging, Department of Biomedical Engineering, Shenzhen University, Shenzhen 518060, Guangdong, China

**Keywords:** metformin, hepatocellular carcinoma, FOXO3, pyroptosis, NLRP3

## Abstract

This study aimed to expand our understanding of metformin (Met) in inhibiting hepatocellular carcinoma (HCC) progression and to investigate its underlying mechanism. Met was administrated to HCC cells at 5, 10, and 20 μM, after which the cell phenotype was evaluated. RNA-seq cluster analysis was used to screen for target genes modulated by Met. Luciferase activity and ChIP assays were performed to detect the effect of FOXO3 on the transcriptional activation of NLRP3. We evaluated the effect of Met and FOXO3 and on the growth of HCC cells *in vivo*. Met inhibited HCC cell proliferation and promoted apoptosis. Met also induced pyroptosis of HCC cells. FOXO3 was significantly upregulated by Met treatment, and FOXO3 activated transcription of NLRP3. Cells after Met treatment together with FOXO3 knockdown have a stronger colony formation and migration ability but a lower apoptosis rate compared to the Met treatment alone group. *In vivo*, Met inhibited HCC tumor growth. The tumors in Met treatment and FOXO3 knockdown group grew faster than in Met treatment group. Thus, Met attenuates HCC cell development by inducing apoptosis and pyroptosis. This effect of metformin is partially dependent on FOXO3 which can activate the transcription of NLRP3.

## INTRODUCTION

Hepatocellular carcinoma (HCC) is the sixth most frequent malignancy worldwide, accounting for almost 90% of primary liver cancers. In addition, it is the third leading cause of cancer-related mortality [1, 2]. Despite significant advances in the diagnosis and treatment of HCC, the five-year survival rate remains under 18% [3]. Accordingly, developing novel strategies in HCC treatment is in great need.

Metformin (Met) is currently one of the first-line drugs for type 2 diabetes mellitus (T2DM) and related diseases such as gestational diabetes, polycystic ovary syndrome, and cystic disease [4]. Activation of AMP-dependent kinase (AMPK) is considered the mechanism of Met against T2DM, which leads to the inhibition of liver glucose production, inhibition of glucose absorption by the stomach and intestines, and insulin sensitization [5].

Although Met was originally introduced as an anti-diabetes medicine, it has other applications. Currently, Met is thought to be potentially useful for treating anxiety [6], vascular disease [7], and air pollution-associated diseases [8]. Moreover, Met may be effective against several type of cancers, including colorectal [9], gastric [10], pancreatic [11], breast [12, 13], lung [14, 15], endometrial [16, 17], ovarian [18, 19], head and neck [20], cervical [21], and bladder cancers [22]. Met can also participate in immunotherapy as an immuno-metabolic adjuvant and take part in anti-angiogenic cancer therapy.

The role of Met on HCC has been widely studied. However, the effect of Met on pyroptosis of HCC has not been reported [23]. Pyroptosis is a novel kind of programmed cell death that differs from necrosis and apoptosis, and has attracted a lot of attention due to its roles in physiological and pathological inflammatory response, oxidative stress, immunity, and cancer progression [24, 25].

In the present study, we performed *in vitro* and *in vivo* studies to confirm the effect of Met on the proliferation, apoptosis, migration, and colony formation of HCC cells. We found that Met induces pyroptosis of HCC cells partially depend on Forkhead box protein O3 (FOXO3) expression and the subsequent activation of NLRP3 transcription. This finding may provide novel strategies or targets for HCC treatment.

## RESULTS

### Met inhibits HCC cell proliferation

**Figure 1:**
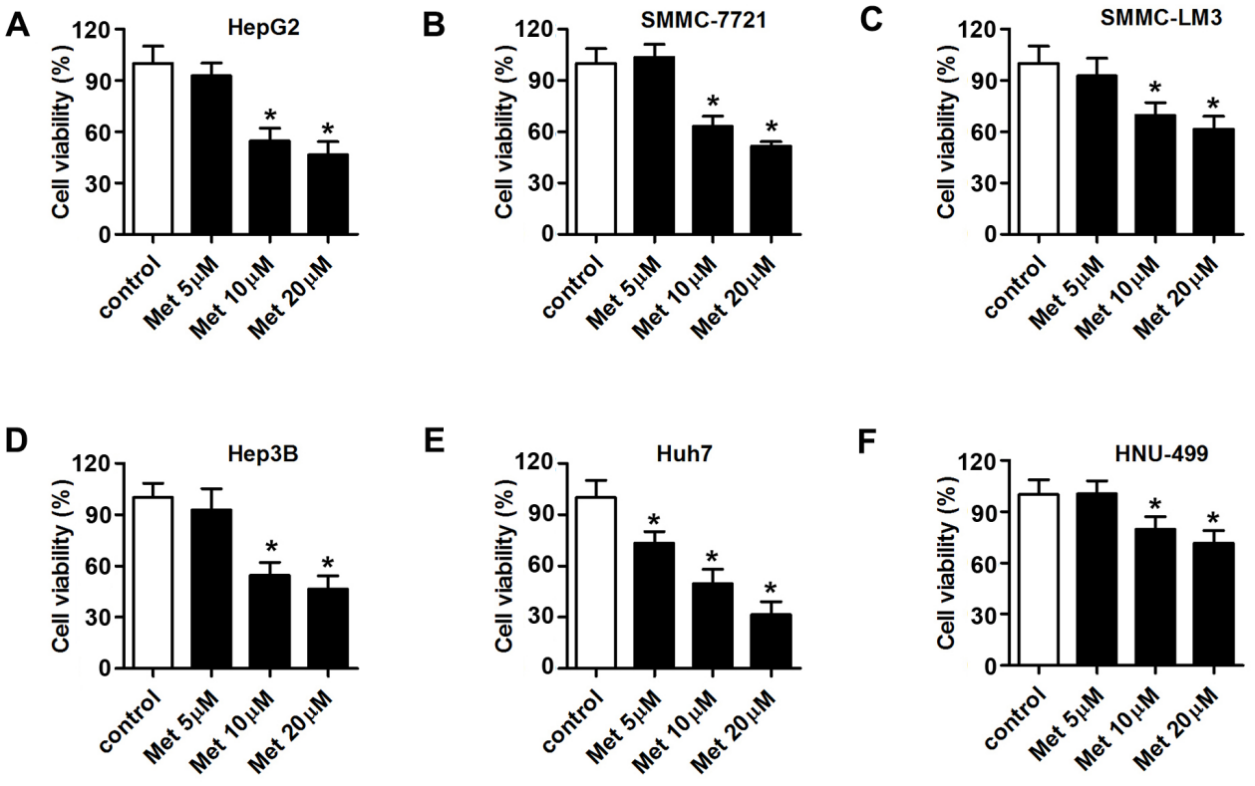
**Met inhibits the proliferation of HCC cells in a dose-dependent manner.** An MTT assay was used to evaluate the proliferation of (**A**) Huh7, (**B**) SMMC-7721, (**C**) SMMC-LM3, (**D**) Hep3B, (**E**) Huh7, and (**F**) HNU-499 cells under different concentrations of Met (5, 10, 20 μM). ^*^P<0.05 vs. control group.

Met exerts anti-tumor effects in various cancers [26, 27]. Thus, we investigated whether Met could suppress HCC cell proliferation. MTT assay results showed that 10 and 20 μM of Met inhibited the proliferation of six types of hepatocellular carcinoma cells, including HepG2, SMMC-7721, SMMC-LM3, Hep3B, Huh7 and HNU-499 cells. The proliferation of Huh7 cells was inhibited by 5 μM of Met (Figure 1A–1F).

### Met induces apoptosis, and inhibits the migration and colony formation of HCC cells

**Figure 2:**
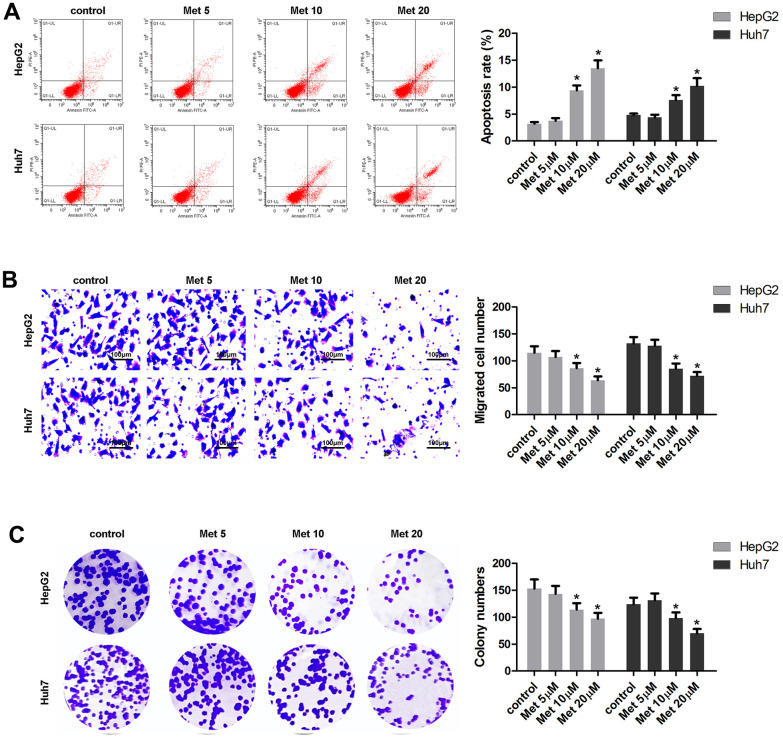
**Met induced apoptosis and inhibited the migration and the colony formation of HepG2 and Huh7 cells in a dose-dependent manner.** (**A**) Annexin V/PI staining and flow cytometry assays were performed to detect the apoptosis. (**B**) A transwell assay was used to evaluate cell migration. (**C**) A colony formation assay was performed to detect the colony formation ability. ^*^P<0.05 vs. control group.

The effects of Met on apoptosis, migration, and colony formation of HepG2 and Huh7 cells are summarized in Figure 2. The results show that 10 and 20 μM of Met induced apoptosis (Figure 2A), and inhibited migration (Figure 2B) as well as colony formation (Figure 2C) of HepG2 and Huh7 cells.

### Met upregulates FOXO3 expression in HCC cells

**Figure 3:**
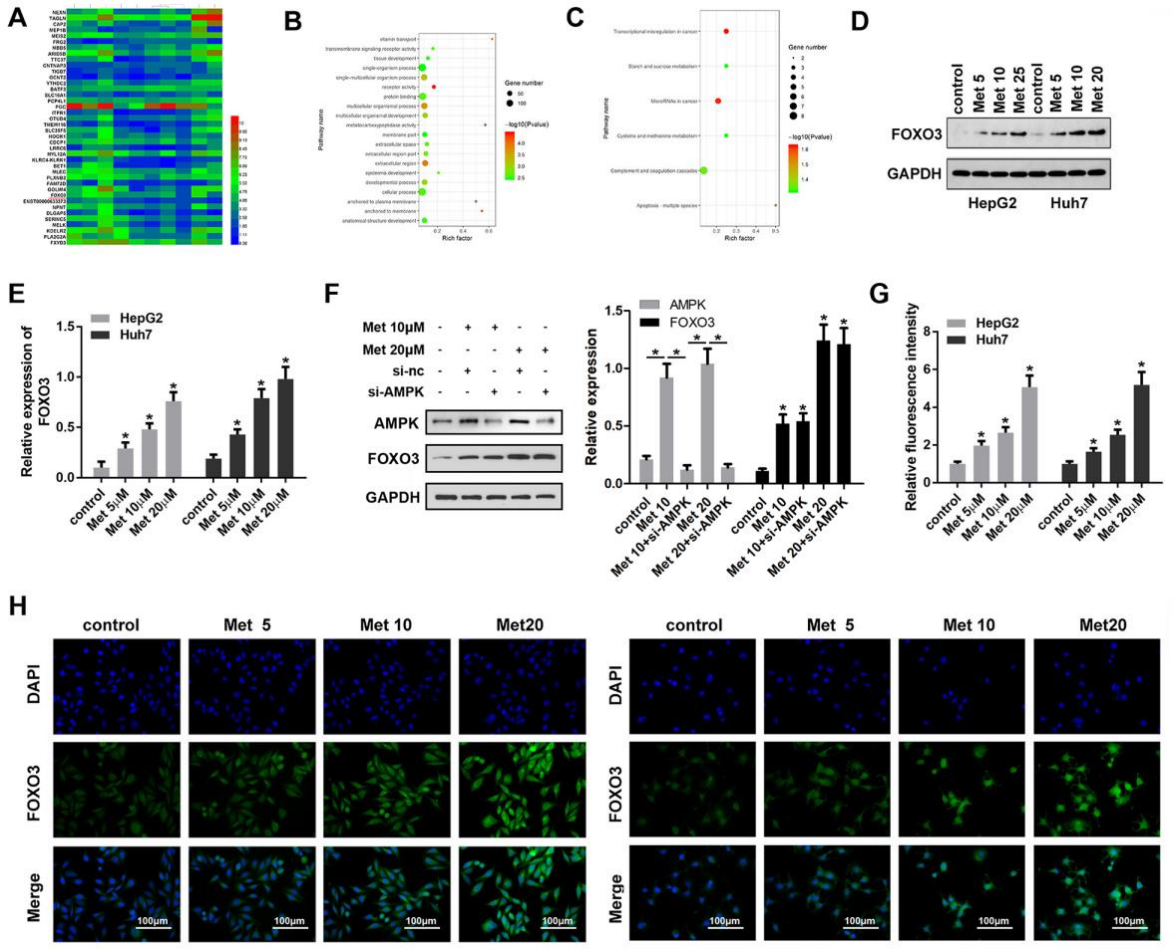
**Met promoted FOXO3 expression level in HepG2 and Huh7 cells.** (**A**) RNA-seq analysis was performed and the dysregulated genes are shown in the heat map. (**B**, **C**) GO/KEGG analysis was carried out to determine clusters of differentially expressed genes with enriched molecular functions. (**D**–**F**) Western blots was performed to evaluate protein expressions (**G**, **H**) immunofluorescence were performed to evaluate the expression of FOXO3 in HepG2 and Huh7 cell lines after treatment with different concentrations of Met. ^*^P<0.05 vs. control group.

To elucidate the molecular mechanism underlying the effect of FOXO3, we performed RNA-seq analysis to screen the dysregulated genes in HCC cells after Met treatment. Then, gene ontology (GO) analysis was used to determine clusters of differentially expressed genes with enriched molecular functions. The heat map in Figure 3A shows the top 20 upregulated and downregulated genes. Among them, FOXO3 was upregulated in HCC cells subjected to Met treatment and enriched for transcriptional misregulation in cancer (Figure 3B, 3C). Subsequently, Western blot (Figure 3D, 3E) results indicated that Met elevated the expression of FOXO3 in a dose-dependent manner. FOXO3 was reported to be regulated by AMPK, thus we performed experiments to see if this regulation also exist in HCC. However, the results showed that Met can promote the expression of AMPK, but silencing of AMPK did not influence the effect of Met on FOXO3 expression (Figure 3F). Immunofluorescence (Figure 3G, 3H) were performed to verify FOXO3 expression in HCC cells after Met treatment. These results confirm that Met elevates the expression of FOXO3 in a dose-dependent manner.

### Knockdown of FOXO3 reverses the effects of Met on HCC cells

**Figure 4:**
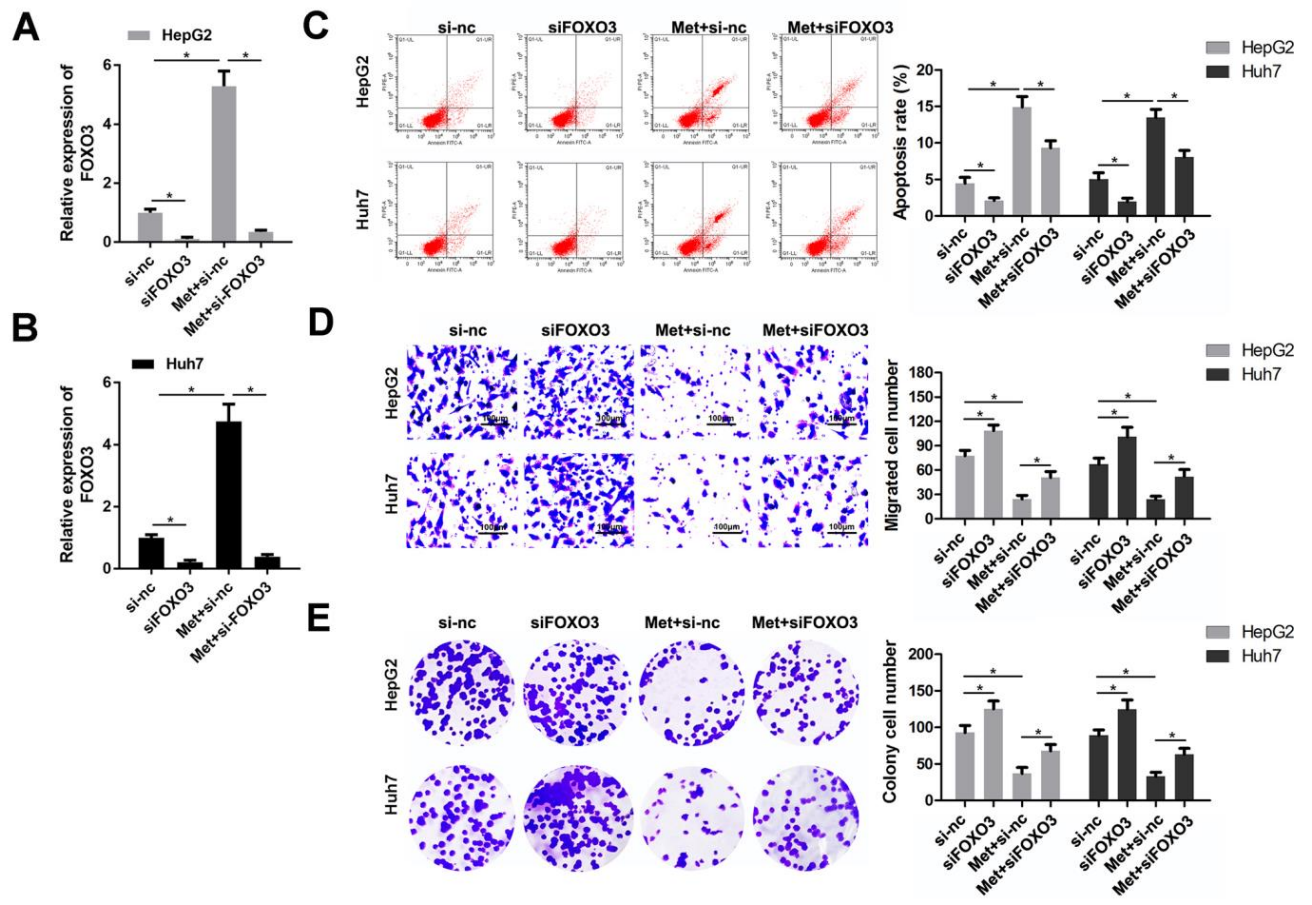
**FOXO3 knockdown reversed the effects of Met on HepG2 and Huh7 cell apoptosis, migration, and proliferation.** (**A**, **B**) qPCR was performed to evaluate the expression of FOXO3 in different groups. (**C**) Annexin V/PI staining and flow cytometry were performed to detect apoptosis. (**D**) A transwell assay was used to evaluate the migration. (**E**) A colony formation assay was performed to detect the colony formation ability. ^*^P<0.05, Met vs. control group, Met + siFOXO3 vs. Met + si-nc group.

As FOXO3 was confirmed to be upregulated by Met treatment, we speculated that FOXO3 plays a critical role in the anti-tumor effect of Met in HCC. Thus, we detected whether FOXO3 knockdown could reverse the effects of Met. First, qPCR results indicated that siFOXO3 notably reduced the expression of FOXO3, while Met promoted FOXO3 expression (Figure 4A, 4B). As expected, HCC cells after Met treatment together with FOXO3 knockdown had a stronger ability of colony formation, migration but a lower apoptosis rate compared to the Met treatment alone group. (Figure 4C–4E). These findings indicate that Met exerts its role partially depend on FOXO3 in HCC cells.

### FOXO3 transcriptionally activates NLRP3

**Figure 5:**
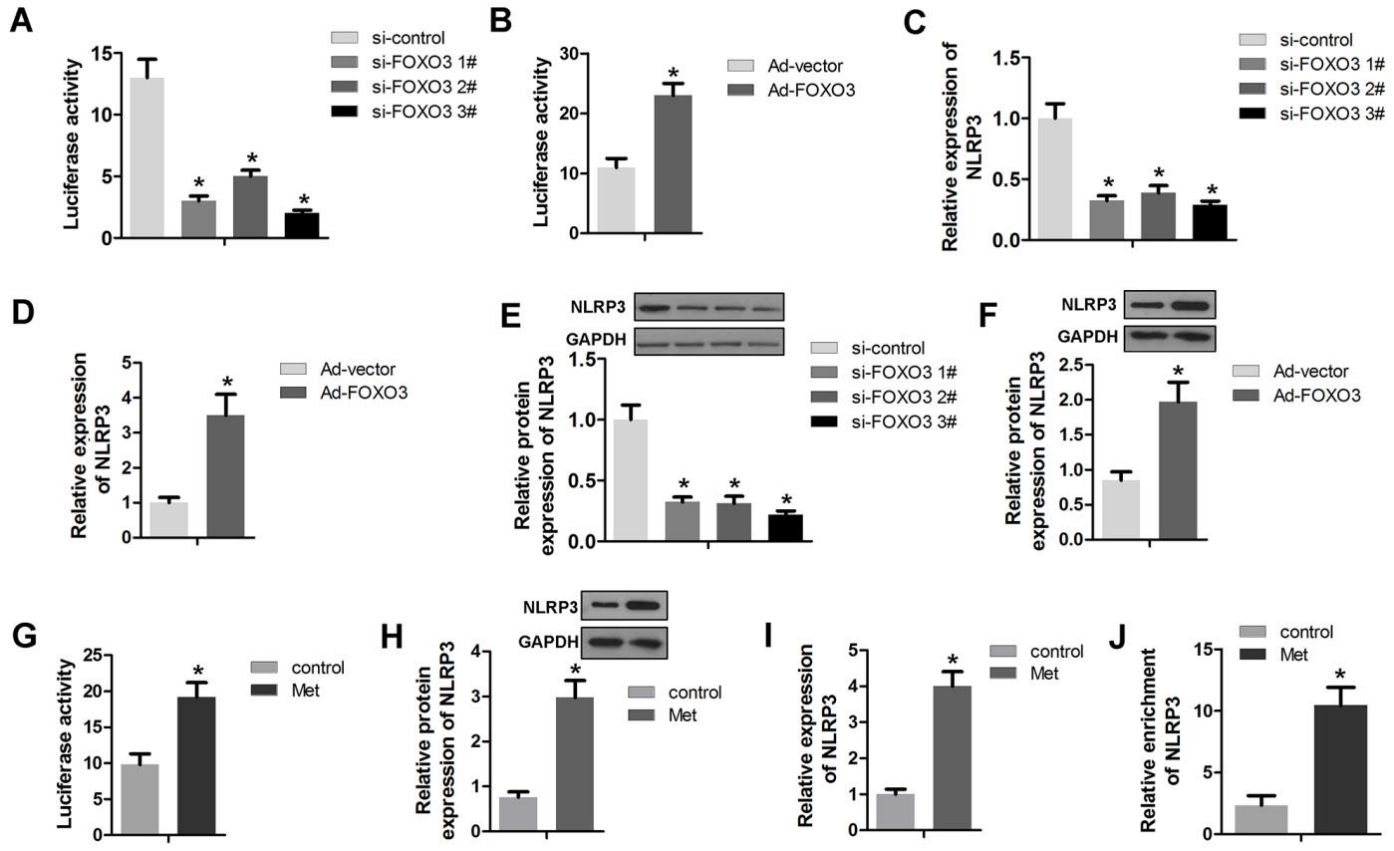
**FOXO3 activates NLRP3 transcription.** (**A**, **B**) A luciferase activity assay was used to determine whether FOXO3 activated NLRP3 transcription. (**C**, **D**) qPCR was used to evaluate the NLRP3 expression after transfection with si-FOXO3 plasmids or vectors overexpressing FOXO3. (**E**, **F**) Western blots were used to evaluate NLRP3 expression after transfection with si-FOXO3 plasmids or vectors overexpressing FOXO3. (**G**) A luciferase activity assay was used to evaluate the transcription activity of NLRP3 after Met treatment. (**H**) Western blot and (**I**) qPCR was performed to evaluate the expression of NLRP3 after Met treatment. (**J**) A ChIP assay detected the interaction between FOXO3 and NLRP3 after Met treatment. *P<0.05 vs. si-nc or vector or control group.

As a transcription factor, FOXO3 plays a critical role in cancer progression via modulating gene activation. Here, we detected the transcriptional activation of FOXO3 on NLRP3. The results show that FOXO3 knockdown could inhibit, while FOXO3 overexpression could promote the transcriptional activity of NLRP3 in HCC cells (Figure 5A, 5B). The RNA and protein expression of NLRP3 were evaluated by qPCR and Western blot, respectively. As revealed by Figure 5C–5F, FOXO3 overexpression promoted RNA and protein expression of NLRP3 while FOXO3 knockdown suppressed NLRP3 expression. Further experiments showed that Met treatment enhanced NLRP3 promoter activity (Figure 5G). Accordingly, Met increased the protein and RNA expression of NLRP3 (Figure 5H, 5I). Finally, a ChIP assay determined that Met promoted the interaction of FOXO3 and the promoter region of NLRP3 (Figure 5J).

### Met induces pyroptosis in HCC cells

**Figure 6:**
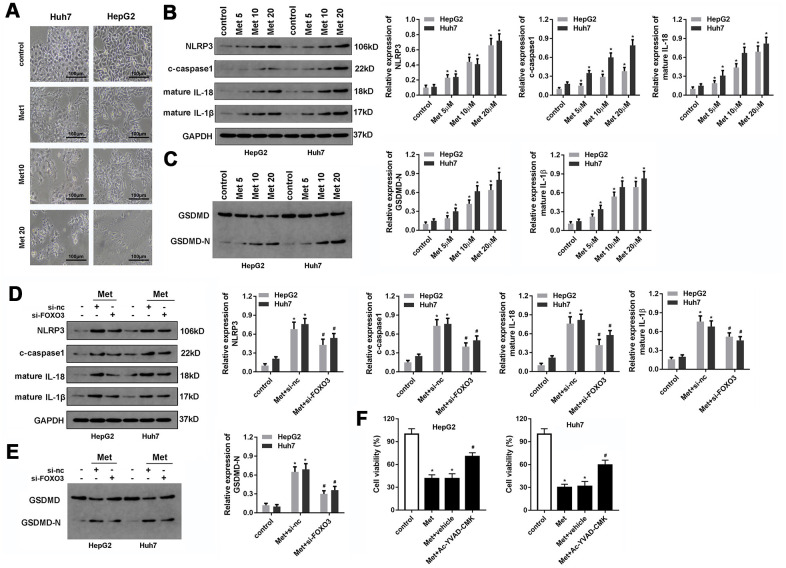
**Met induced pyroptosis in HepG2 and Huh7 cells.** (**A**) Morphologic observation of the HepG2 and Huh7 cell lines after treatment with different concentrations of Met. (**B**, **C**) Western blot was performed to evaluate the expression level of pyroptosis-related proteins NLRP3, cleaved-caspase1, IL-1β, IL-18, and GSDMD after Met treatment. (**D**, **E**) Western blot was performed to evaluate the expression level of pyroptosis-related proteins NLRP3, cleaved-caspase1, IL-1β, IL-18, and GSDMD after co-treatment with different concentrations of Met and si-FOXO3. (**F**) MTT was used to evaluate the cell viability of HepG2 and Huh7 cells. ^*^P<0.05 vs control group, ^#^P<0.05 vs Met+vehicle group.

Met was reported to induce pyroptosis in esophageal carcinoma. We sought to determine whether Met inhibits HCC progression in ways beyond apoptosis or necrosis. Interestingly, pyroptosis was observed in HepG2 and Huh7 cells after Met treatment (Figure 6A). To confirm this finding, we evaluated the expression of pyroptosis-related proteins. As expected, 10 and 20 μM of Met promoted the expression of NLRP3, c-caspase1, IL-1β, and IL-18 (Figure 6B). In addition, Met promoted the cleavage of GSDMD into GSDMD-N (Figure 6C). To verify whether FOXO3 was involved in the regulation of pyroptosis, we used si-FOXO3 to knockdown FOXO3 and detected pyroptosis-related markers. These results indicate that FOXO3 knockdown reverses the effect of Met on HCC pyroptosis (Figure 6D, 6E). The rescue experiments indicated that the block of pyroptosis using caspase1 inhibitor Ac-YVAD-FMK reversed the effect of Met on the cell viability of HCC cells (Figure 6F).

### The expression levels of pyroptotic markers including NLRP3 and IL-1β in HCC

**Figure 7:**
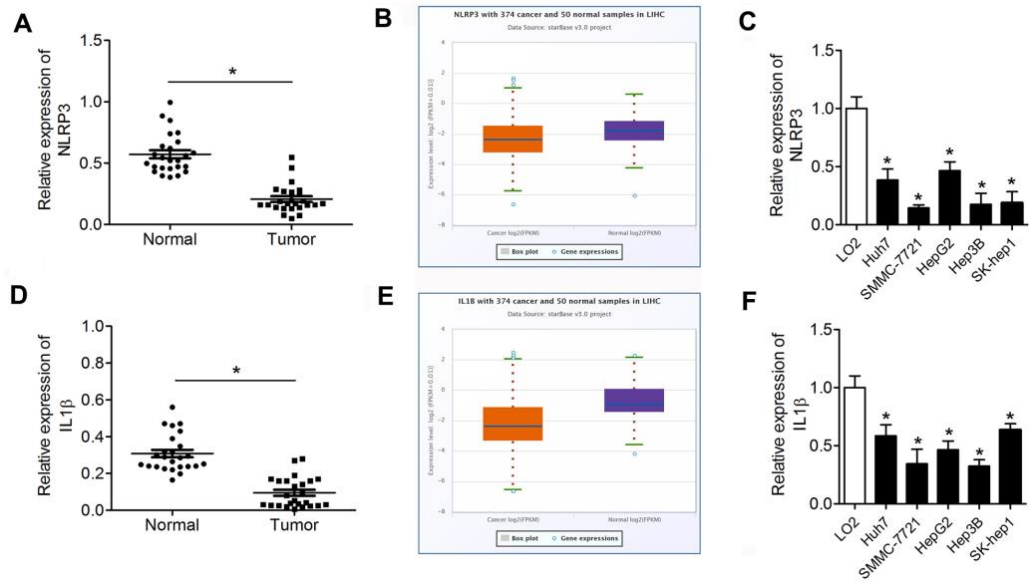
**NLRP3 and IL-1β are downregulated in HCC tissues and cell lines.** (**A**, **D**) qPCR was performed to evaluate NLRP3 and IL-1β expression in HCC tumor tissues and paracancerous tissues. (**B**, **E**) The expression data of NLRP3 and IL-1β in the starBase dataset. (**C**, **F**) Western blot was performed to evaluate the expression of NLRP3 and IL-1β in HCC cell lines. ^*^P<0.05 vs. normal or LO2 group. HCC, hepatocellular carcinoma.

We detected the expression of pyroptotic markers NLRP3 and IL-1β in the HCC using qPCR and analyzed the expression data through the starBase dataset (http://starbase.sysu.edu.cn/). The results are presented in Figure 7. NLRP3 was notably downregulated in HCC tumor tissues and cell lines compared with normal tissues and LO2 cells (Figure 7A–7C). Similarly, IL-1β was downregulated in HCC tumor tissues and cell lines compared with normal tissues and LO2 cells (Figure 7D–7F).

### Met inhibits tumor growth partially depend on FOXO3

**Figure 8:**
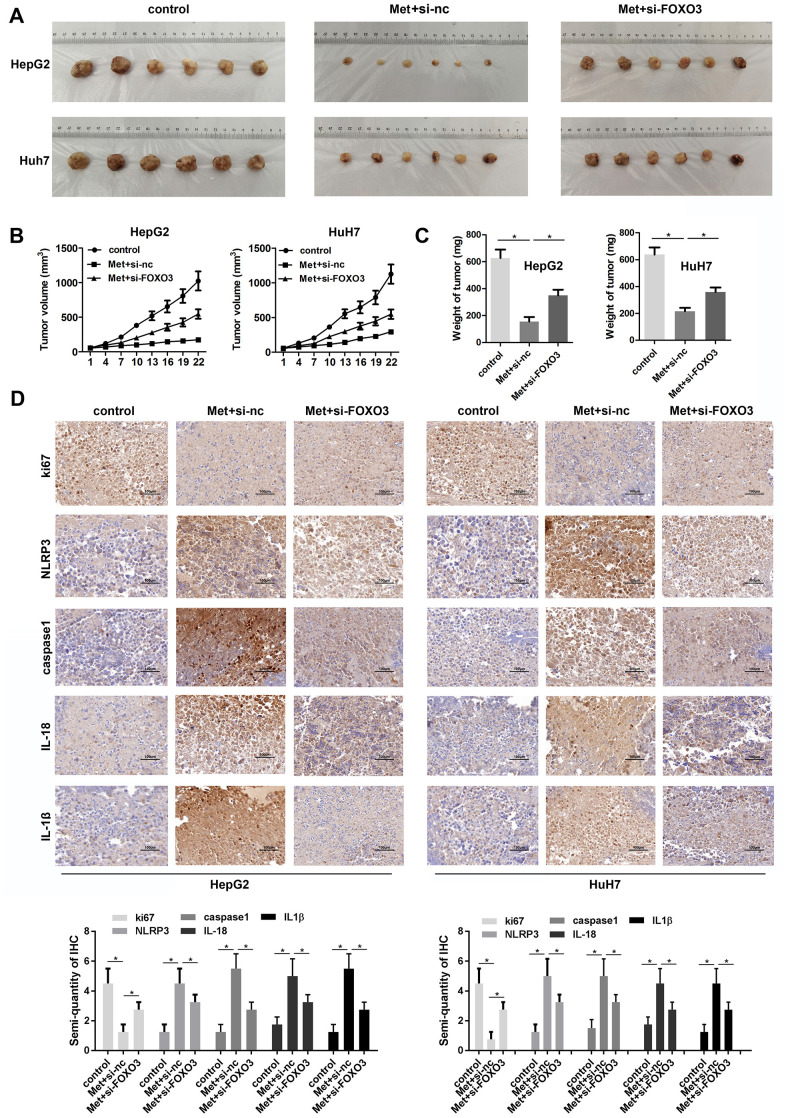
**Met inhibits HCC cell growth *in vivo*.** (**A**) Image of tumors in each group. (**B**) Growth curves of the tumors in each group. (**C**) Tumor weights in the different groups. (**D**) IHC was used to evaluate the expression of Ki-67, NLRP3, caspase1, IL-1β, and IL-18. ^*^P<0.05, Met vs. control group, Met + si-FOXO3 vs. Met+si-nc group.

To further investigate the anti-tumor effect of Met on HCC, we performed *in vivo* animal studies. Met treatment significantly inhibited tumor growth and reduced tumor weight of HCC. The tumors in Met treatment and FOXO3 knockdown group grew faster than that in Met treatment group. (Figure 8A–8C). IHC staining showed that Ki-67 was downregulated in the tumor tissues of the Met treatment group. The expression of pyroptosis markers including NLRP3, caspase1, mature IL-1β, and IL-18 were upregulated after Met administration. FOXO3 knockdown partially reversed these effects of Met (Figure 8D). In summary, Met inhibits tumor growth partially depend on FOXO3.

## DISCUSSION

In this study, we found that Met inhibits the proliferation, migration, and colony formation of HepG2 and Huh7 cells and promotes their apoptosis. In addition, Met induced pyroptosis and inhibited tumorigenesis of HCC partially depend on FOXO3 expression. Suppression of FOXO3 can partially revers the effects of Met.

Met, originally developed to treat T2DM, is involved in the progression of various cancers, including hepatocellular carcinoma. In the present study, we confirmed the anti-tumor effect of Met in HCC cells. Interestingly, we found that FOXO3 was upregulated in HCC cells after Met treatment.

FOXO3 belongs to the forkhead transcription factors of the FOXO subfamily (FOXOs), and is involved in cell cycle arrest, apoptosis, and the oxidative stress response [28, 29]. As a critical transcription factor, FOXO3 participates in the progression of numerous cancers [30, 31].

Met was previously shown to suppress radiation-induced skin injuries by modulating the expression of FOXO3 through PIK3r1 [32]. In addition, Met can activate FOXO3 to promote the differentiation of stem-like glioma-initiating cells into nontumorigenic cells [33]. Moreover, Met also has an anti-proliferative effect in breast cancer via activating FOXO3 [34]. However, the effect of Met on FOXO3 expression has not been reported in HCC. We demonstrated that Met promoted FOXO3 expression in HepG2 and Huh7 cells.

FOXO3 functions as an apoptosis trigger through the upregulation of genes critical for cell death including Bim and PUMA [35], or the downregulation of anti-apoptotic proteins such as FLIP [36]. FOXO3 is widely considered as a “suppressor of cancers”. Our results support the theory that FOXO3 promotes cell death and inhibits cell proliferation. The limitation here is the lack of research on the downstream genes of FOXO3 related to cell proliferation.

Pyroptosis is a new kind of programmed cell death, different from necrosis and apoptosis. During the process of pyroptosis, immune cells recognize foreign danger signals within themselves, release pro-inflammatory cytokines, swell, burst, and die [24]. Pyroptosis can be considered as a process between necrosis and apoptosis. We found that the pyroptosis markers IL-1β and NLRP3 were significantly downregulated in HCC, indicating the potential role of pyroptosis in HCC progression. In this study, we also observed that Met induced pyroptosis through FOXO3. The connection between FOXO3 and pyroptosis was reported previously [37, 38], but there has been no report integrating Met, FOXO3, pyroptosis, and cancer. This study shows that Met promotes FOXO3 expression and subsequent activation of NLRP3 transcription, which results in pyroptosis of HCC. According to previous studies [39–41], Met regulates FOXO3 through the AMPK pathway. We found that Met could promote the expression of AMPK, but the role of Met on FOXO3 expression is independent of AMPK. NLRP3 is regarded as an intracellular signaling molecule that is implicated in the inflammatory response and development of human diseases. Formation of the NLRP3 inflammasome composed of NLRP3, an apoptosis-associated speck-like protein (ASC), and caspase-1, which is the key step of pyroptosis. Future work should be performed to elucidate the precise mechanism.

However, there are some limitations in our study. First, apoptosis-specific caspases should be inhibited to prove that the cell death observed in HCC is pyroptosis instead of apoptosis. Hence, in the future, we will block caspase 1 and other apoptosis-related factors to prevent cell death like targeting FOXO3. In another study [32], Kim et al. claimed that Met could downregulate FOXO3 via PIK3r1. However, we found that Met could upregulate FOXO3. Moreover, we found that Met elevated the expression of FOXO3 and FOXO3 are crucial for the progression of liver cancer. FOXO3 must play a role mediating the effect of Met, however, much more work should be carried out to elucidate the precise mechanism. We plan to perform *in vivo* studies using old mice in future work. Collectively, we will further investigate the effect of PIK3r1 on the regulatory mechanisms between Met and FOXO3.

In summary, we demonstrated that Met exerts anti-tumor and pyroptosis-inducing effects on HCC cells which expands our understanding of Met pharmacology. Mechanistically, Met promoted the expression of FOXO3 and activated NLRP3 transcription. These findings may provide novel therapeutic targets or treatment strategies against HCC.

## MATERIALS AND METHODS

### Clinical samples

25 tumor and the adjacent normal tissues were collected from hepatocellular carcinoma patients hospitalized at Jinling Hospital and stored at -80° C. All patients did not undergo chemotherapy or radiotherapy treatment previously. This study was approved by the Ethics Committee of Nanjing University. The participants in this study provided informed consent. All experiments were performed following the Helsinki declaration.

### Cell culture

The hepatocellular carcinoma cells HepG2, SMMC-7721, SMMC-LM3, Hep3B, Huh7, and HNU-499 were purchased from ATCC (Maryland, USA). Cells were cultured in Dulbecco's Modified Eagle's Medium (DMEM, Gibco, USA) supplemented with 10% fetal bovine serum (FBS, Gibco), 100 U/mL penicillin, and 100 mg/mL streptomycin (Invitrogen, CA, USA) at 37° C and the presence of 5% CO_2_.

### Transfection and stable cell line generation

The adenoviruses for silencing or overexpressing FOXO3 and the controls plasmids were established by Genepharma (Shanghai, China). In brief, the full-length cDNA fragment was amplified by PCR. Then, a recombinant adenoviral vector expressing FOXO3 was cloned and ligated into cells using the One Step Cloning Kit (Vazyme Biotech, China).

Lentiviral plasmids were transfected into HEK-293 cells using virus-packing plasmids to produce the lentivirus. Then, the stable cells expressing high levels of si-FOXO3 and the negative control were selected in the presence of 500 μg/mL puromycin for four weeks. Finally, cell transfection was carried out using the Lipofectamine 2000 reagent (Thermo Fisher Scientific, USA) following the manufacturers’ protocol.

### MTT assay

The cells in the logarithmic phase were resuspended at the density of 5×10^4^ cells/ml and seeded into the 96-well plates at 100 μl /well. 24 h later, 0.5% MTT dye (10 μl, Sigma, USA) was added to each well of the plate, then the plate was cultured in the incubator at 37° C for 4 h. The supernatant was removed and DMSO added to each well. The absorbance values were determined at the wavelength of 490 nm with a microplate reader (Bio-Rad, USA).

### Colony formation assay

The cells were resuspended at a density of 1×10^3^ cells/ml. Subsequently, 100 μl cell suspension was seeded into each well of the 12-well plates, and the cells were cultured for 14 days. At the end of the incubation period, the cells were washed twice with PBS, fixed with 4% paraformaldehyde (Invitrogen, USA) and stained with 0.1% crystal violet. Three independent experiments were performed, and cell clusters containing > 50 cells were counted.

### qRT-PCR

Cells were mixed with TRIzol® reagent (Invitrogen, Carlsbad, USA.) to extract the total RNA. Reverse transcription were carried out using an RNA PCR Kit (Takara, Dalian, China). qPCR was carried out using a iQ SYBR Green Super MixTo kit (Bio-Rad, USA) to detect gene expression. PCR reaction was performed using an iCycler iQ system. All primers were designed and synthesized by Nanjing Genscript Biotech Co., Ltd., GAPDH and U6 were used as internal controls for the mRNAs and miRNAs respectively. Fold changes of the indicated genes were calculated using 2-ΔΔCt method.

### Western blot

Total protein was extracted from HepG2 and Huh7 cells by lysis buffer for 30 min, and was quantified using the BCA protein Assay Kit (Beyotime, Shanghai, China). 10% SDS-PAGE was used to separate 40 μg of the protein for 1.5 h at 120 V. Subsequently, the separated protein was transferred onto the PVDF membranes (Millipore) for 2 h. The membranes were then blocked with 5% skim milk for 2 h followed by incubating with primary antibodies overnight at 4° C. The primary antibodies used were as follows: rabbit anti-FOXO3a (ab109629, 1:1000, Abcam, Shanghai, China); rabbit anti-NLRP3 (ab263899, 1:1000, Abcam, Shanghai, China); rabbit anti-cleaved caspase 1 (PA5-38099, 1:1000, Thermo Fisher Scientific, Inc., Waltham, USA); rabbit anti-IL-18 (ab207324, 1:200, Abcam, Shanghai, China); rabbit anti-IL-1β (ab200478, 1:1000, Abcam, Shanghai, China); rabbit anti-GAPDH (ab8245, 1:1000, Abcam, Shanghai, China); rabbit anti-GSMDM (ab209845, 1:1000, Abcam, Shanghai, China); and rabbit anti-GSDMD-N (ab215203, 1:1000, Abcam, Shanghai, China). After three washes with tris-buffered saline with Tween-20 (TBST), the blots were incubated at room temperature for 90 min with HRP-conjugated secondary antibody (ab7090, 1:1000, Abcam, Shanghai, China). GAPDH was used to normalize protein expression levels. At the end, and the bands were visualized using an ECL plus reagent (Pierce, Rockford, IL, USA) and a LAS 4000 charge-coupled device (Fujifilm, USA).

### Flow cytometry

Apoptotic cells were stained by Annexin V-FITC Apoptosis Detection Kit (Solarbio, Beijing, China). 5 μl of the Annexin V-FITC was added to each well of the 6-well plate, and the cells were resuspended at the density of 1×106 ml. Cell Apoptosis was detected using BD FACSCalibur system (Becton Dickinson, NJ, USA).

### Luciferase reporter assay

The 1800 bp sequences containing NLRP3 promoter potential binding sites were constructed into dual-luciferase reporters (Promega, USA). HEK-293 cells were transfected with pGL3 reporter carrying the sequence of NLRP3 along with an si-control or si-FOXO3 using Lipofectamine 2000 regents (Invitrogen, CA, USA). pRL-CMV plasmid was transfected as internal control. Luciferase activities were measured 24 h post transfection using a dual-luciferase reporter kit (Promega). The relative luciferase activity was normalized to the activity of Renilla luciferase.

### Chromatin immunoprecipitation assay (ChIP)

An EZ ChIP kit (Millipore, CA, USA) was used to perform the ChIP assay. HepG2 and Huh7 cells were trypsinized and washed with PBS. After centrifugation, the cells were crosslinked in 1% formaldehyde at 37° C for 10 min. Chromatin was sonicated into fragments which was then incubated with the antibody against FOXO3. The enrichment of the DNA fragments by the antibodies were assessed using qPCR assay.

### Immunostaining

Cells for immunofluorescent staining were treated with 4% paraformaldehyde for 20 min followed by permeabilizing using 0.5% Triton X-100 for 15 min. Then, cells were incubated in 3% BSA for 30 min for the blockage. Thereafter, cells were stained with the primary antibodies at 4° C overnight. After washing with PBS for 3 times, the membranes were stained with an Alexa Fluor-labelled secondary antibody (Invitrogen, USA) for 2 h. Finally, the blots were stained with DAPI for 5 min (Beyotime, Shanghai, China). After which, the cells were observed under a fluorescence microscope (Leica, Wetzlar, Germany).

### Animal studies

Fifty BALB/c nude male mice (6-week-old, weighing 18-20 g) were obtained from the Vital River Laboratory Animal Technology Co. They were randomly divided into six groups. HepG2 and Huh7 cells stably overexpressing si-FOXO3 or its negative control (si-nc) were subcutaneously injected at a dose of 1×10^6^ into the mice's flank region. Met, at the dose of 20 mg/kg, was administered to each group of mice. The tumor size was detected every 2 days with a calliper. All the mice were sacrificed at the 28^th^ day and the tumors were removed. A part of the tissue was placed in 10% formalin for histological analysis and the remaining tissue was frozen at -80° C. The animal study were approved by the Ethics Committee of Jinling Hospital, Medical School of Nanjing University.

### Immunohistochemistry (IHC)

The 5 μm sections were deparaffinized and rehydrated in graded alcohol. Endogenous peroxidase blockage was carried out using 3% H_2_O_2_. Thereafter, antigen repair was performed using 10 mM of citrate buffer and heating at 95° C. Subsequently, sections were incubated with the primary antibodies overnight at 4° C, then incubated with with the HRP-labelled secondary antibody at room temperature for 2 h. A 3,3'-diaminobenzidine (DAB) detection system (Dako, Denmark) was used to detect primary antibodies according to manufacturer’s protocol. The quantification of the IHC results was performed using a semi-quantitatively method: score 0 means no staining or the area of stained field <33%, score 1 means moderate staining or the area of stained field <33%, score 2 means strong staining or the area of stained field 33-66%; The two numbers were multiplied to be the final score.

### Statistical analysis

SPSS 19.0 statistical software was used for data analysis. All data was represented as the mean ± standard deviation (SD) (x±s). The least significant method (LSD) method in the one way analysis of variance (ANOVA) was used for comparison between two groups. P<0.05 was considered as statistically significant. P < 0.01 was statistically very significant.
